# Novel Mesoporous Flowerlike Iron Sulfide Hierarchitectures: Facile Synthesis and Fast Lithium Storage Capability

**DOI:** 10.3390/nano7120431

**Published:** 2017-12-06

**Authors:** Quanning Ma, Qianyu Zhuang, Jun Liang, Zhonghua Zhang, Jing Liu, Hongrui Peng, Changming Mao, Guicun Li

**Affiliations:** College of Materials Science and Engineering, Qingdao University of Science and Technology, Qingdao 266042, China; maquanning@hotmail.com (Q.M.); 18354210108@163.com (Q.Z.); junlianglj@163.com (J.L.); zhang_zhua@126.com (Z.Z.); qnma@mails.qust.edu.cn (J.L.); penghongrui@qust.edu.cn (H.P.)

**Keywords:** iron sulfide, three-dimensional flowerlike structure, hierarchical pore structure, lithium ion batteries

## Abstract

The 3D flowerlike iron sulfide (F-FeS) is successfully synthesized via a facile one-step sulfurization process, and the electrochemical properties as anode materials for lithium ion batteries (LIBs) are investigated. Compared with bulk iron sulfide, we find that the unique structural features, overall flowerlike structure, composed of several dozen nanopetals and numerous small size iron sulfide particles embedded within the fine nanopetals, and hierarchical pore structure features provide signification improvements in lithium storage performance, with a high-rate discharge capacity of 779.0 mAh g^−1^ at a rate of 5 A g^−1^, due to effectively alleviating the volume expansion during the lithiation/delithiation process, and shorting the diffusion length of both lithium ion and electron. Especially, an excellent cycling stability are achieved, a high discharge capacity of 890 mAh g^−1^ retained at a rate of 1.0 A g^−1^, suggesting its promising applications in lithium ion batteries (LIBs).

## 1. Introduction

Currently, one of the major challenges is providing high-efficient, low-cost, and environmentally benign electrochemical energy conversion and storage devices to power increasingly various applications, ranging from numerous portable electronic devices to electric vehicles (EVs) or plug-in hybrid electric vehicles (PHEVs) [1,2,3]. Among various energy storage system, Lithium ion batteries (LIBs) and supercapacitors are at the frontier. Meantime, lithium ion batteries have attracted enough attention, due to their high energy/power densities, and good rate performance [4,5,6,7]. The future applications will require a higher energy/power density at a high rate for LIBs, however, the mostly used commercial anode material, graphite, only delivered a low theoretical capacity of 372 mAh g^−1^ [8]. Therefore, further breakthroughs in material hold the key to the next generation of LIBs.

Nanostructure materials are becoming increasingly important, and have attracted great attention, due to the unusual electrical and mechanical properties endowed by confining the dimensions of such material, and due to the combination of surface and bulk properties to the whole behavior [5,9,10,11]. There are many advantages in nanostructured material over their bulk counterparts. The size reduction into the nano-scale leads to a higher electrode/electrolyte contact area, making higher charge/discharge rates, and shortened Li^+^ and electronic transport path lengths (permitting operation with low Li^+^ and electronic conductivity or higher power) [6,12,13,14,15,16]. Also, the nanostructure can make a better accommodation of stresses and strains associated with the crystal unit cell changes during lithium ion insertion/removal, improving the structural stability and cycling life. Moreover, its interface between electrolyte and electrode materials is strengthened, leading to a higher lithium ion flux compared with bulk structure. Meanwhile, in nanostructured materials, new reactions with electrolyte, not possible in bulk materials, has occurred. The increased reactivity with electrolytes, because of the high surface area resulting from size reduction, may adversely affect the electrochemical performance of LIBs [5,17,18,19].

Recently, considerable interest has focused on materials with three-dimensional (3D) hierarchical nano/microstructures, due to the 3D architecture providing a highway for the diffusion of both electrolyte and electron, which can facilitate efficient energy storage. Among these, materials with 3D flowerlike structure have been widely used as anodes in LIBs. For example, Wang et al. prepared flowerlike Co_9_S_8_/Co_1−x_S@NC, with a discharge capacity of 813 mAh g^−1^ delivered at a current density of 2.0 A g^−1^ [20]. Hu et al. fabricated flowerlike MoS_2_@C composites, delivering a discharge capacity of 697 mAh g^−1^ at a current density of 5 A g^−1^ [21]. Li et al. synthesized the flowerlike NiCo_2_O_4_, delivering a discharge capacity of 420 mAh g^−1^ at a rate of 2 A g^−1^ [22]. Therefore, the aforementioned facts confirmed that the three-dimensional (3D) flowerlike hierarchical nano/microstructure was effective for improving the high-rate performance and cycling stability.

However, iron sulfide based materials with three-dimensional (3D) flowerlike hierarchical nano/microstructure were rarely reported. Besides, iron sulfide based material possessed a high energy/power density and abundant reserves in the earth, having also attracted considerable attention as alternative promising anode materials for LIBs [23,24]. Based on the above considerations, we develop a powerful strategy for fabricating a 3D flowerlike structure (nano/micropetals connected to each other through the center), including iron sulfide (FeS) nanoparticles embedded in nano/micropetals (3D F-FeS), through synthesizing the Fe-based precursors, and the subsequent in-situ sulfide treatment. The unique nanostructure and hierarchical pore structure of F-FeS can effectively alleviate the volume expansion during the charge/discharge process, and shorten the diffusion length of both lithium ion and electron. Benefiting from its merits, F-FeS exhibits superior high-rate performance (779.0 mAh g^−1^ at a high rate of 5 A g^−1^) and cycling performance (890.0 mAh g^−1^ retained after 100 cycles at a rate of 1 A g^−1^), further highlighting its tremendous potential for commercial application.

## 2. Materials and Methods Results

### 2.1. Synthesis of 3D Flowerlike Fe-Based Precursor

All the chemicals were directly used without further purification. In a typical synthesis with some modifications, 25 mM (1.2 g) FeCl_3_·6H_2_O, 250 mM (2.7 g) urea, and 124 mM (7.2 g) tetrabutylammonium bromide were dissolved into 180 mL ethylene glycol (EG) in a 250 mL round-bottom flask. The red solution was obtained after chemicals completely dissolved. Then, the solution was gradually heated to refluxing temperature (ca. 185 °C) under stirring. After 12 min, a yellow precipitate appeared, and then the mixture turned yellow-green immediately. After 8 min, the mixture turned completely green, indicating the formation of the 3D flowerlike hierarchical nanostructure. After refluxing for 30 min, the reaction was ceased, and the mixture was cooled at ambient condition. After cooling to room temperature, the green precipitate was collected, and rinsed thoroughly with ethanol three times, before thoroughly drying at 60 °C in air.

### 2.2. Synthesis of Uniform 3D F-FeS Nanostructure

Briefly, the obtained precursor and sulfur (with a weight ratio 1:3) were loaded into two separate alumina boats, then transferred to a tube furnace for sulfurization at 600 °C for 5 h, with a heating rate of 1 °C min^−1^ under Ar atmosphere. Finally, the FeS with 3D flowerlike nanostructure were obtained. As comparison, one sample was annealed under air atmosphere at 450 °C for 5 h with a ramp of 3 °C min^−1^, before the sulfurization process. The sulfurization process was the same as mentioned earlier. After that, bulk FeS (B-FeS) was synthesized.

### 2.3. Materials Characterization

The crystal structures were characterized by X-ray diffraction analysis (XRD Rigaku D-max-γA XRD with Cu K_α_ radiation, λ = 1.54178 Å). Field-emission scanning electron microscopy (FE-SEM; JM 6700F), and transmission electron microscope (TEM; JEM-2100Plus, JEOL Ltd., Tokyo, Japan) were used to investigate the morphology and microstructure of the as-obtained F-FeS and B-FeS. Thermogravimetric (TG) analysis was measured on a TG apparatus (NETZSCH TG 209-F1, NETZSCH-Gerätebau GmbH., Selb, Germany) in the temperature range of 25–900 °C at a ramp of 10 °C min^−1^ in air. The Brunauer–Emmett–Teller (BET) method was utilized to investigate the specific area by N_2_ adsorption–desorption isotherms. To further characterize the chemical composition, XPS analysis was performed with X-ray photoelectron spectroscopy (Thermo ESCALAB 250XI, Thermo Fisher Scientific Inc., Waltham, MA, USA). Raman spectra was also performed to investigate the existence of carbon.

### 2.4. Electrochemical Measurements

Electrochemical performances were tested in CR2032-type coin cell at room temperature. For preparing electrode, the active material was mixed with carbon black (Super-P) and poly(vinyl difluoride) (PVDF) binder with a mass ratio of 70:20:10, then dissolved in *N*-methyl-pyrrolidone (NMP) to produce electrode slurry. The slurry was uniformly pasted onto pure copper foil current collector. After drying at 120 °C in a vacuum oven for 6 h, the Cu foil coated active material was used as working electrode, with metallic lithium foil as the counter electrode and separated by a Celgard 2500 membrane separator. The cells were assembled in a glove box filled with pure argon. After shelving for 12 h, the galvanostatic discharge–charge measurements were conducted on LAND CT2001A battery tester at various current densities over a voltage range of 0.0–3.0 V (vs. Li^+^/Li). Cyclic voltammetry (CV) curves and electrochemical impedance spectroscopy (EIS) were measured on the Autolab PGSTATN302N electrochemical workstation (Metrohm, Herisau, Switzerland). Cyclic voltammetry (CV) tests were carried out at a scanning rate of 0.1 mV s^−1^ from 0.0 to 3.0 V. For the electrochemical impedance spectroscopy (EIS), the measurements were performed over a frequency range from 100 KHz to 10 mHz with 10 mHz. This section may be divided by subheadings. It should provide a concise and precise description of the experimental results, their interpretation, as well as the experimental conclusions that can be drawn.

## 3. Results and Discussion

**Figure 1:**
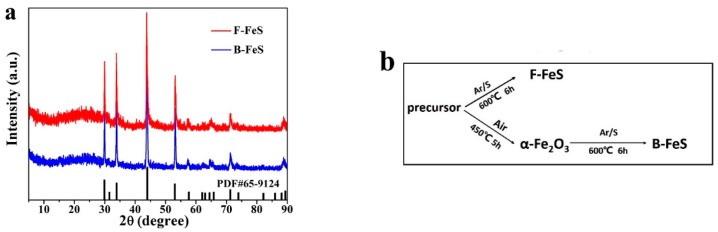
(**a**) XRD patterns of the as-prepared F-FeS and B-FeS; (**b**) Schematic illustration of the calcination process.

The unique 3D F-FeS nanostructure has been successfully synthesized via a simple one-step sulfurization reaction at 600 °C under Ar atmosphere. Particularly, 3D flowerlike Fe-based precursors were prepared according to previous literature, with some modifications; X-ray diffraction pattern (XRD; Figure S1a) showed the diffraction peaks similar to those of Fe-based precursors in the literature [25]. For obtained B-FeS material, it has been annealed in air before sulfurization, and the diffraction peaks of the product annealed in air were in good agreement with α-Fe_2_O_3_ (Hematite, JCPDS 80-2377), as shown in Figure S1b. After sulfurization, the corresponding X-ray diffraction pattern (XRD; Figure 1a) confirms the existence of FeS pure phase in both F-FeS and B-FeS. All diffraction peaks can be exclusively attributed to hexagonal FeS (JCPDS 65-9124). The simple calcination procedure was as illustrated in Figure 1b.

**Figure 2:**
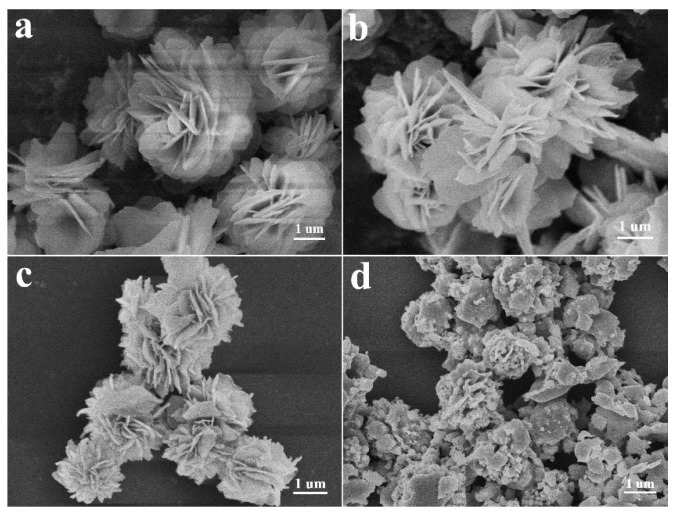
Typical SEM image of as-prepared Fe-based precursor (**a**); α-Fe_2_O_3_ (**b**); F-FeS (**c**); and B-FeS (**d**).

**Figure 3:**
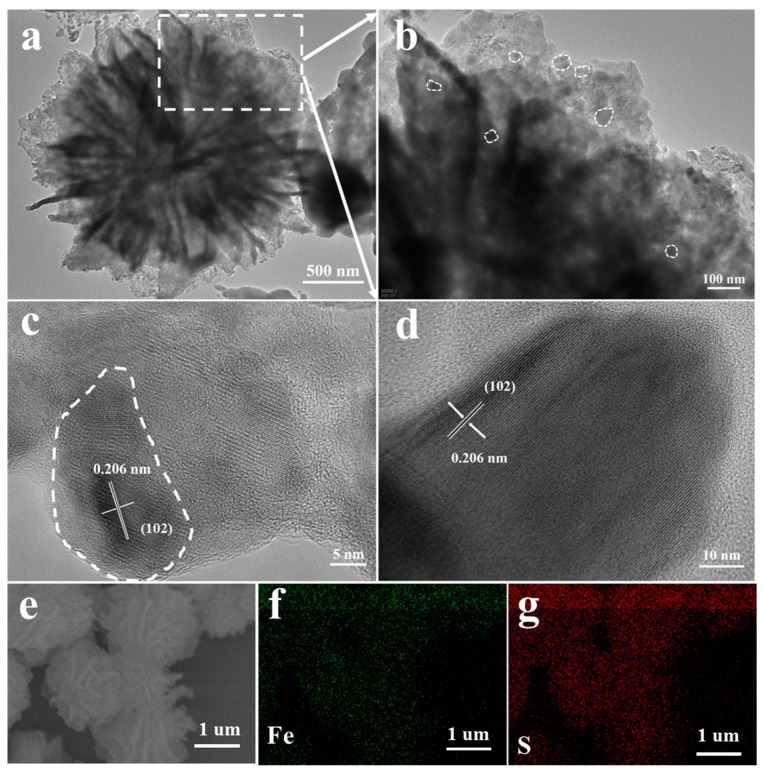
(**a**,**b**) TEM images of the as-prepared 3D F-FeS; (**c**,**d**) HRTEM images of the 3D F-FeS and B-FeS; (**e**–**g**) EDS elemental mapping showing the homogenous distribution of Fe and S elements in 3D F-FeS nanostructure.

The morphology and microstructure of the as-prepared products were investigated by field-emission scanning electron microscopy (FE-SEM) as illustrated in Figure 2. SEM image of the Fe-based precursor composed of uniform flowerlike architectures, approximately 2–3 μm in diameter, is shown in Figure 2a. The detailed morphology of the flowerlike nanostructure is also exhibited clearly in the SEM image, which shows that the entire nanostructure of the architecture is constructed from several dozen fine nanopetals. The fine nanopetals were approximately 60–80 nm thick, and 1–2 μm wide, and connected to each other through the center forming the 3D flowerlike structure. Its low-resolution SEM image exhibits uniform distribution of the as-obtained 3D flowerlike Fe-based precursors (Figure S2). Figure 2b shows the morphology of flowerlike α-Fe_2_O_3_ obtained after the annealing process in air. The flowerlike architecture is perfectly inherited, and the morphology of nanopetals has no significant structural breakage or collapse, as shown in Figure S3. After the procedure of sulfurization, for the B-FeS, the structure and morphology changed hugely, the flowerlike architecture has been pulverized, and no nanopetal structure could be clearly observed (Figure 2d), instead, collapsing into irregular particles (Figure S5). However, the 3D F-FeS sustained the original structure and morphology which directly sulfurized using the precursor, revealing its excellent structural stability, which is crucial to the electrochemical performances (Figure 2c). The surface of these fine nanopetals becomes rougher than that of the precursors (Figure S4). The structural difference may be ascribed to the existence of organic molecules before sulfurization, serving as structural stabilizer during the process of sulfurization. Transmission electron microscopy (TEM) and high-resolution TEM (HRTEM) images provide further insight into the detailed nanostructure of the as-prepared 3D F-FeS and B-FeS. As shown in Figure 3a, the 3D flowerlike nanostructure of F-FeS consisted of several dozen fine nanopetals, consistent with SEM images. In a high resolution (Figure 3b), many small-sized FeS particles with a diameter of 20–40 nm were observed in the fine nanopetals. Meanwhile, the porous features formed by the loose stack of small-size FeS particles can also be clearly elucidated by the sharp contrast between the light and dark areas, beneficial for restricting volume expansions during the lithium intercalation/deintercalation process. Moreover, the pores may serve as lithium ion transportation channels. And the connected fine nanopetals may work as an electron conduction skeleton. The unique nanostructural features created a large contact area with electrolyte, and reduced the diffusion path length for lithium ion, promoting the fast transportation of lithium ions and electrons, which are beneficial for the improvement of cycling performance [14]. Figure 3c,d shows the high-resolution TEM images of 3D F-FeS and B-FeS, where the same regular lattice fringes with an interplanar distance of approximately 0.206 nm were observed, corresponding to the (102) plane of hexagonal FeS, in agreement with the analysis results of XRD patterns. The EDS elemental mapping images exhibited in Figure 3e–g confirmed the existence and homogeneous distribution of Fe and S elements in the nanostructure of 3D F-FeS. A similar phenomenon can also be seen in elemental mapping images of B-FeS (Figure S6a–c).

**Figure 4:**
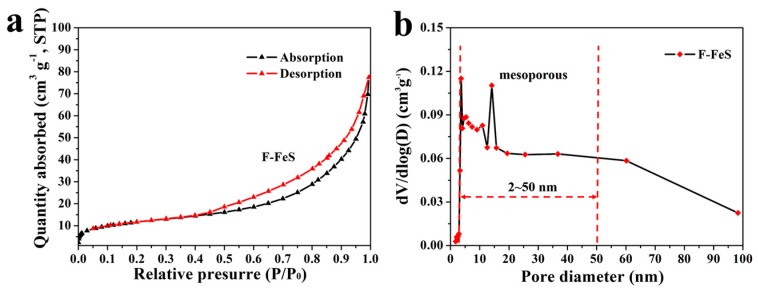
(**a**) Nitrogen adsorption–desorption isotherms and (**b**) pore size distribution curve of 3D F-FeS.

In order to further investigate the porosity properties, N_2_ physisorption measurements were carried out. As shown in Figure 4a, the as-prepared 3D F-FeS achieves a higher Brunauer–Emmett–Teller (BET) surface area of around 40.9 m^2^ g^−1^, than that of the B-FeS (13.3 m^2^ g^−1^) (Figure S7). The pore size distribution curve of 3D F-FeS further indicated the porous nanostructure with abundant mesopores in the range of 3–20 nm. The hierarchical pore structure and larger surface area can effectively promote the penetration of electrolytes in electrodes, further improving the electrochemical performance for LIBs [20].

**Figure 5:**
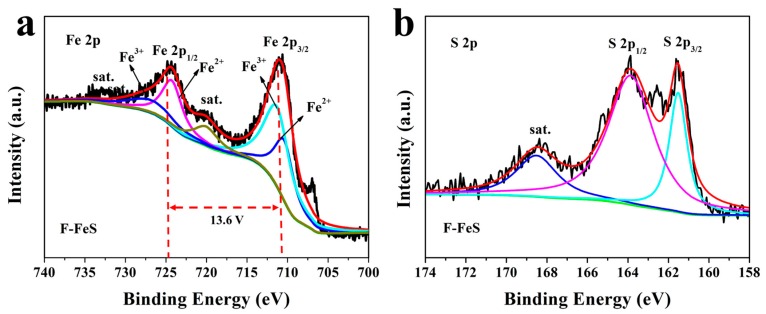
XPS spectra for the as-prepared 3D F-FeS nanostructure: (**a**) Fe 2p and (**b**) S 2p spectra.

Besides, X-ray photoelectron spectroscopy (XPS) was performed to further investigate the information on the surface chemical compositions and valence states of the 3D F-FeS. Figure 5a shows the high-resolution XPS spectrum of the Fe 2p core-level. Peaks observed at ~710.99 and 724.59 eV can be attribute to Fe 2p_3/2_ and Fe 2p_1/2_, respectively, indicating the existence of Fe^2+^ in 3D F-FeS. Two satellite peaks were observed at ~719.25 and 732.78 eV. The signal centered at ~706.78 eV was maybe attributed to a little of metallic Fe. Additionally, peaks observed at ~711.27 and 726.74 eV were ascribed to Fe^3+^, due to the Fe^2+^ partly oxidizing in air on the surface of material [26,27]. As shown in the high-resolution XPS spectrum (Figure 5b), three peaks at ~161.49, 163.93, and 168.41 eV were detected. The peaks at ~161.49 and 163.93 eV corresponded to S 2p_3/2_ and S 2p_1/2_, respectively, the characteristic peaks of FeS, whereas another peak at ~168.41 eV probably corresponded to oxidized group (SO*_x_*), due to the sulfur species being partly oxidized in air on the surface of material [28]. The similar high-resolution XPS spectrums of B-FeS were shown in Figure S9a,b.

**Figure 6:**
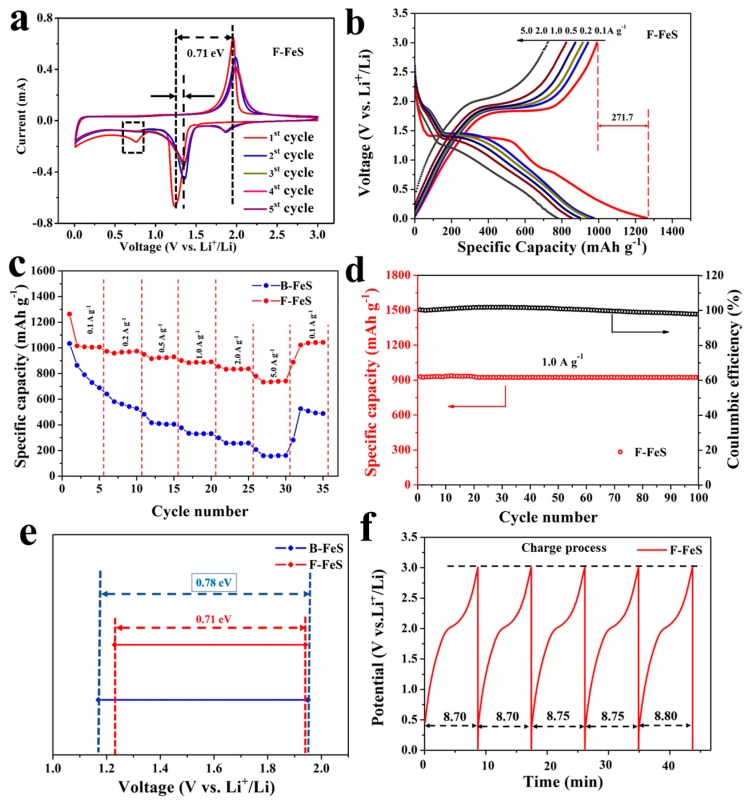
Kinetics investigation of the as-prepared 3D F-FeS nanostructure: (**a**) corresponding galvanostatic discharge/charge at various current densities and (**b**) rate performance; (**c**) long-term cyclic performance at the current density of 1.0 A g^−1^; (**d**) CV curves at a scan rate of 0.1 mV s^−1^; (**e**) potential difference between cathodic peak and anodic peak in cyclic voltammetry profile of the first cycle; (**f**) corresponding charge curves.

In order to evaluate the potential use of 3D F-FeS nanostructures as anode for LIBs, as-prepared materials were assembled into half-cells and evaluated by a series of electrochemical measurements. Cyclic voltammetry (CV) was applied to investigate the redox reactions taking place in 3D F-FeS and B-FeS. Figure 6a shows typical CV curves for the 3D F-FeS. During the first cycle of the CV curves, two cathodic peaks at 1.23 and 0.75 V and one anodic peaks at 1.94 V are observed. During the discharge process, the sharp reduction peak at 1.23 V was related to the conversion reaction between FeS and Li (Equation (1)) [29], and a peak at 0.75 V was attributed to the formation of the solid-electrolyte interface (SEI) layer, which gradually disappeared with cycling [30,31]. During charge process, the oxidation peak at 1.94 V corresponded to the oxidation of Fe to Li_2−*x*_FeS_2_ (0 < *x* < 2) (Equation (2)). The shape of CV curves changes after the first cycle, suggesting that the redox reactions undergo changes in the subsequent cycles. In the subsequent lithiation cycles, the cathodic peaks appearing at 1.86 V was attributed to the step-by-step formation of Li_2_FeS_2_ from Li_2−*x*_FeS_2_ (0 < *x* < 2) phase. The anodic peaks appearing in the subsequent cycles were contributed to the delithiation process from Li_2−*x*_FeS_2_ (0 < *x* < 2) to Li_2_FeS_2_. The reaction process between Li_2−*x*_FeS_2_ (0 < *x* < 2) and Li_2_FeS_2_ is reversible [32,33,34]. From the second cycle onwards, in the CV curves, the cathodic peaks were shifted to ≈1.34 V from 1.23 V. The phenomenon of increased voltage after the process of initial lithiation was already widely reported for many conversion electrodes, which mainly related to stress/strain and structure changes produced during the process of initial conversion reactions [35]. The anodic peaks were still localized at ≈1.94 V. Almost overlapped CV curves in the subsequent cycles indicate the excellent reversibility and cycling stability of 3D F-FeS, which makes 3D F-FeS that achieve superior electrochemical performance. In comparison, the CV curves of B-FeS are shown in Figure S10, exhibiting similar peaks. However, the curves were not well-overlapped, indicating the poor reversibility and cycling stability of B-FeS during lithiation/delithiation process.

(1) $$2\mathrm{FeS}+2\mathrm{Li}+2e^{-}\to \mathrm{Fe}+{\mathrm{Li}}_{2}S$$

(2) $$\mathrm{Fe}+{\mathrm{Li}}_{2}S-x{\mathrm{Li}}^{+}-xe^{-}\to {\mathrm{Li}}_{2-x}{\mathrm{FeS}}_{2}$$

Figure 6b performs the galvanic discharge–charge voltage profile of 3D F-FeS at various current densities of 0.1, 0.2, 0.5, 1.0, 2.0, 5.0 A g^−1^. The 3D F-FeS delivered an initial discharge capacity of 1262.9 mAh g^−1^, and a charge capacity of 991.2 mAh g^−1^ at a current density of 0.1 A g^−1^, giving an irreversible capacity loss of 271.7 mAh g^−1^ and a Coulombic efficiency of 78.5%. The initial capacity loss can be attributed to the inevitable formation of solid-electrolyte interface (SEI) layer and initial irreversible lithium consumption. At the current densities of 0.2, 0.5, 1.0, 2.0 A g^−1^, it delivered discharge capacities of 973.3, 949.3, 900.7, 854.9 mAh g^−1^. At a high current density of 5 A g^−1^, 3D F-FeS still delivered a discharge capacity of 779.0 mAh g^−1^, much higher than that of B-FeS (206 mAh g^−1^, Figure S11), indicating that the unique 3D flowerlike hierarchical structure provided excellent reversible capacity and potential for application to LIBs. At the current densities of 0.1, 0.2, 0.5, 1.0, 2.0, and 5.0 A g^−1^, the B-FeS delivered discharge capacities of 1038.5, 640.2, 488.0, 378.3, 300.0, and 208.2 mAh g^−1^, respectively. Compared with 3D F-FeS electrode, the capacity of B-FeS electrode decreased rapidly with the current density increasing, exhibiting more inferior lithium storage properties than 3D F-FeS electrode at high current densities. Table S1 exhibits the results in comparison with FeS-based electrodes reported in the literature. Meanwhile, as shown in the first discharge curve of 3D F-FeS, a long voltage plateau at around 1.42 V was observed, in addition to a small slope between 0.88 and 0.80 V, contributing to the formation of Li_2_S and Fe as well as the solid-electrolyte interface (SEI) layer, respectively. In the subsequent discharge curves, the slope between 0.88 and 0.8 V disappeared, a small slope showed around 1.9 V. These phenomena are consistent with the CV analysis. Compared with B-FeS, the 3D F-FeS exhibited outstanding high-rate capability as an anode material in LIBs (Figure 6c). When the current density was recovered to 0.1 A g^−1^ from 5 A g^−1^, the 3D F-FeS electrode can still return to the original value. Figure 6d depicts the cycle performance of 3D F-FeS at a current density of 1.0 A g^−1^. The capacity of around 890.0 mAh g^−1^ was retained after 100 cycles, with a Coulombic efficiency of nearly 100%, suggesting the superior cycling performance. The poor cycling performance of B-FeS was shown in Figure S12. The unique flowerlike structure with small-size FeS particles was crucial for improving the cycling stability. Moreover, according to the Figure 6e, the unique 3D flowerlike structure of F-FeS provided faster transportation of lithium ion than bulk FeS, due to the smaller polarization phenomenon in the first CV curves. Figure 6f showed the voltage curves along charging time at a current density of 5.0 A g^−1^, the shape and charging time of five curves is similar, suggesting the superior reversibility of 3D-F-FeS at a high rate.

**Figure 7:**
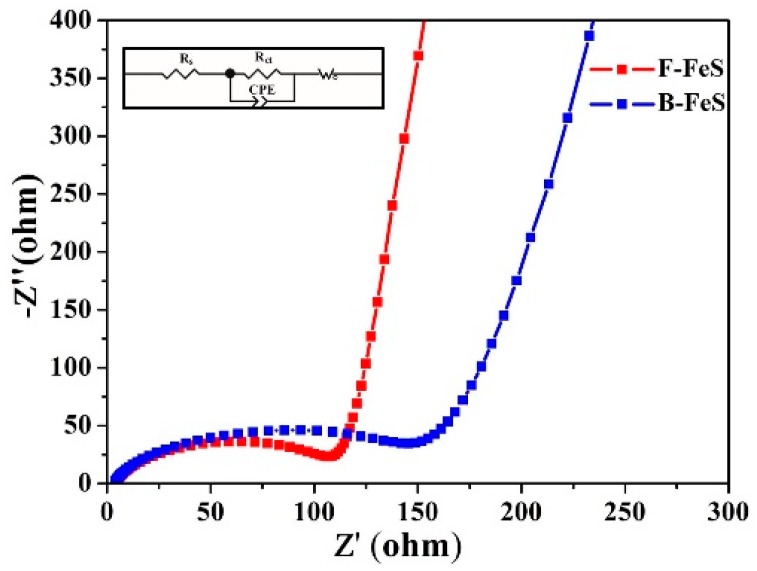
Electrochemical impedance spectra and equivalent circuit of 3D F-FeS and B-FeS nanostructures electrodes before cycling. A sine wave with amplitude of 10.0 mV over the frequency range from 100 kHz to 10 mHz.

To further understand interfacial properties of the 3D F-FeS with respect to the B-FeS electrode, electrochemical impedance spectroscopy (EIS) measurements were taken. As shown in Figure 7, the Nyquist plots of 3D F-FeS and B-FeS both showed a semicircle in high-frequency region and a sloping straight line in low-frequency region. The equivalent circuit model is also exhibited in Figure 7 inset to represent the internal resistance [36,37]. The *R*_s_ is ohmic resistance related to the Li^+^ transport in the electrolyte. The semicircle in the high-frequency region corresponded to the charge transfer impedance (*R*_ct_) and constant phase element (CPE) between electrode/electrolyte interface [38,39,40]. Clearly, the diameter of the semicircle for 3D F-FeS nanostructure electrode in the high-frequency region is much smaller than that of B-FeS electrode, implying that the 3D F-FeS nanostructure electrode possesses a favorable charge transfer compared with B-FeS electrode. The result also confirmed that the unique 3D flowerlike nanostructure improved the cycling stability of 3D F-FeS electrode. Moreover, according to the sloping straight line at low-frequency region, the lithium-diffusion process of 3D F-FeS nanostructure electrode is much easier than that of B-FeS electrode, further validating that the flowerlike structure benefits the lithium-diffusion process of the electrode. The kinetic difference between 3D F-FeS and B-FeS electrodes were further studied by modeling AC impedance spectra based on the equivalent circuit [41], and Table S2 listed the fitted impedance parameters. It can be seen that the charge-transfer resistance R_ct_ of 3D F-FeS electrode is 97.89 Ω, which is much smaller than that of B-FeS (114.7 Ω). This fact, once again, validated that the unique flowerlike structure and small size FeS particles enhanced rapid electron transport during the process of lithiation/delithiation reactions, improving the electrochemical performances significantly. The EIS spectra after 100 cycles is shown in Figure S13. Clearly, the diameter of the semicircle for 3D F-FeS nanostructure electrode in the high-frequency region is still much smaller than that of B-FeS electrode, possessing lower charge transfer impedance (≈131.5 Ω) than that of B-FeS electrode (≈320.1 Ω) and better kinetic performances.

Additionally, Figure S14 showed the SEM image of 3D F-FeS electrode at full-charge state (3.0 V) after cycling at 0.1 A g^−1^, and the 3D F-FeS material still sustains the original flowerlike framework, exhibiting its structure stability during the lithiation and delithiation process. Meantime, compared with 3D F-FeS material before cycling (Figure 2c and Figure S4), the difference in the surface of active materials could be observed clearly, due to the confined SEI formation to the surface of the nanopetals after cycling. The spatially confined and stable SEI formation is helpful to enhance the cycling stability of 3D F-FeS electrode.

The superior lithium storage performance, including high specific capacity, excellent high rate capability and cycling stability, of the 3D F-FeS electrode could be assigned to the following structural advantages: (1) the small size iron sulfide particles in the nanopetals could shorten the diffusion path length of lithium ions and alleviate the volume strain during the lithiation/delithiation process, which effectively promote the high rate capability and cycling stability; (2) the fine nanopetals assembled by small size iron sulfide particles possess dual-channel structure, greatly promoting the fast transportation for both lithium ions and electrons, which make the iron sulfide active material fully utilized; (3) the 3D flowerlike structure facilitated the penetration of electrolyte into electrode, due to its hierarchical pore structure and high specific surface area.

## 4. Conclusions

In summary, the 3D flowerlike iron sulfide (F-FeS) with high phase purity were facilely synthesized through one-step sulfurization of Fe-based precursors, and used as anode material in LIBs. The overall flowerlike structural features composed of several dozen nanopetals were perfectly inherited after sulfurization process and numerous small size iron sulfide particles embedded within the fine nanopetals. Benefiting from the unique structural features and hierarchical pore structure, the 3D flowerlike iron sulfide (F-FeS) electrodes manifested impressive lithium storage properties with a high-rate discharge capacity of 779.0 mAh g^−1^ at a current density of 5 A g^−1^. In particular, an excellent cycling stability can be achieved at a current density of 1.0 A g^−1^ up to 100 cycles, even retaining a high capacity of 890 mAh g^−1^, confirming the 3D flowerlike iron sulfide as a promising anode material for LIBs.

## Supplementary Materials

The following are available online at http://www.mdpi.com/2079-4991/7/12/431/s1, Figure S1: XRD patterns: Fe-based precursor (a) and flowerlike α-Fe_2_O_3_ (b), Figure S2: High-resolution SEM image of flowerlike Fe-based precursor, Figure S3: The high-resolution SEM image of α-Fe_2_O_3_ in the rectangular region shown in Figure 2b, Figure S4: The high-resolution SEM image of 3D F-FeS in the rectangular region shown in Figure 2c, Figure S5: The high-resolution SEM image of 3D F-FeS in the rectangular region shown in Figure 2d, Figure S6: EDS elemental mapping showing the homogenous distribution of Fe and S elements in B-FeS nanostructure, Figure S7: Nitrogen adsorption–desorption isotherms of B-FeS, Figure S8: Pore size distribution curve of as-prepared B-FeS, Figure S9: XPS spectra for the as-prepared B-FeS nanostructure: (a) Fe 2p and (b) S 2p spectra, Figure S10: CV curves of B-FeS at a scan rate of 0.1 mV s^−1^, Figure S11: Corresponding galvanostatic discharge/charge at various current densities for B-FeS electrode, Figure S12: Long-term cyclic performance of B-FeS at the current density of 1.0 A g^−1^, Figure S13: Electrochemical impedance spectra and equivalent circuit of 3D F-FeS and B-FeS nanostructures electrodes after cycling. A sine wave with amplitude of 10.0 mV over the frequency range from 100 kHz to 10 mHz, Figure S14: SEM image of 3D F-FeS electrode at full-charge state (3.0 V) after cycling at a current density of 0.1 A g^−1^, Table S1: Comparison with previous reports, Table S2: Impedance parameters obtained using equivalent circuit model for 3D F-FeS and B-FeS nanostructure electrodes.
