# Supplementary material for: Novel Mesoporous Flowerlike Iron Sulfide Hierarchitectures: Facile Synthesis and Fast Lithium Storage Capability
Source: Nanomaterials (Basel). 2017 Dec 6;7(12):431. doi: 10.3390/nano7120431 (PMC5746921; doi:10.3390/nano7120431)
Supplement: Supplementary file 1 [file nanomaterials-07-00431-s001.pdf]

Novel Mesoporous Flowerlike Iron Sulfide Hierarchitectures: Facile Synthesis and Fast Lithium Storage Capability

Quanning Ma, Qianyu Zhuang, Jun Liang, Zhonghua Zhang, Jing Liu, Hongrui Peng, Changming Mao*, Guicun Li*

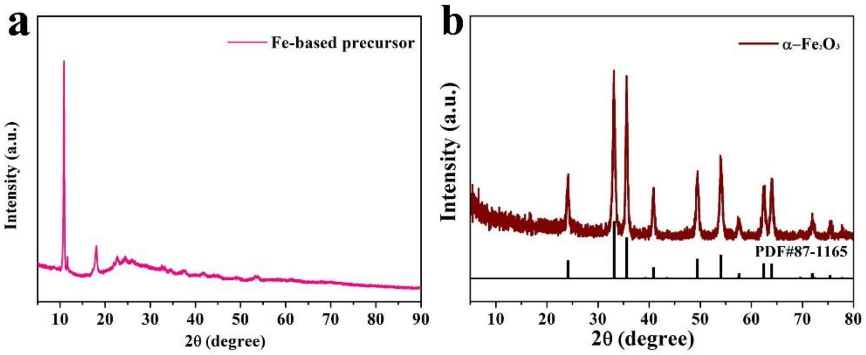

Figure S1. XRD patterns: Fe-based precursor (a) and flowerlike α-Fe_2O_3 (b).

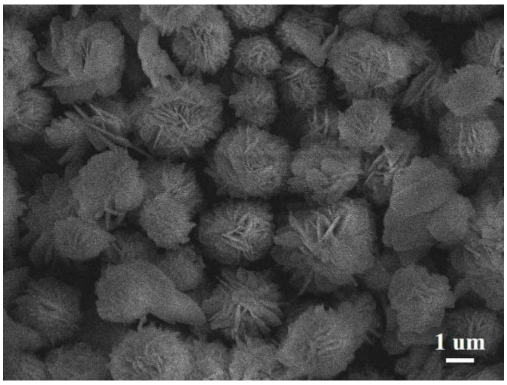

Figure S2. High-resolution SEM image of flowerlike Fe-based precursor.

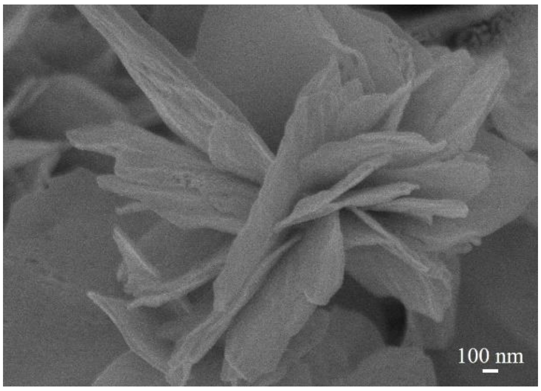

Figure S3. The high-resolution SEM image of α-Fe_2O_3 in the rectangular region shown in Figure 2b.

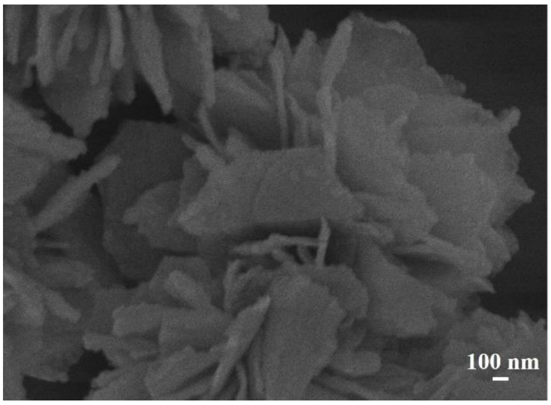

Figure S4. The high-resolution SEM image of 3D F-FeS in the rectangular region shown in Figure 2c.

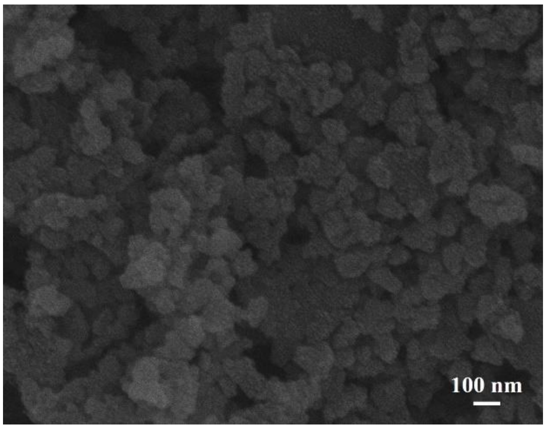

Figure S5. The high-resolution SEM image of 3D F-FeS in the rectangular region shown in Figure 2d.

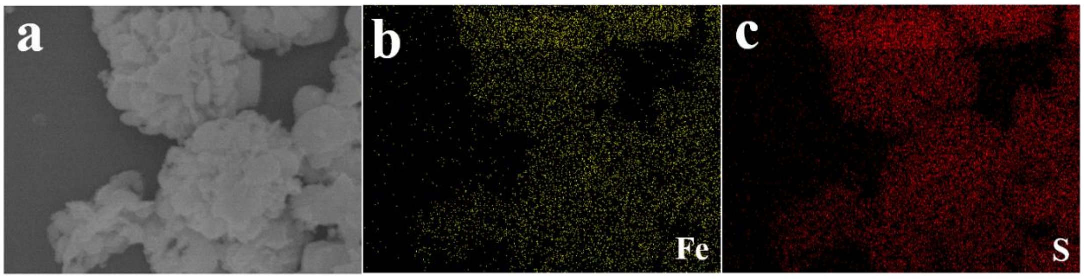

Figure S6. EDS elemental mapping showing the homogenous distribution of Fe and S elements in B-FeS nanostructure

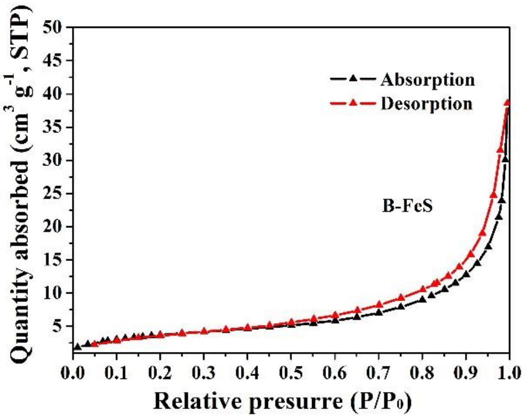

Figure S7. Nitrogen adsorption–desorption isotherms of B-FeS.

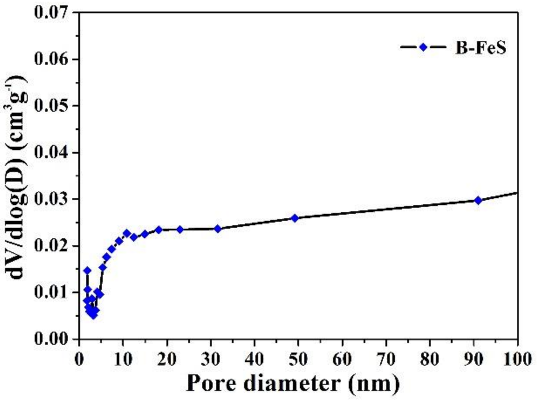

Figure S8. Pore size distribution curve of as-prepared B-FeS.

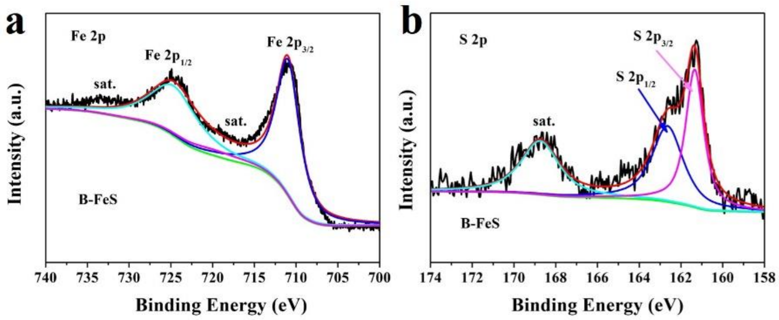

Figure S9. XPS spectra for the as-prepared B-FeS nanostructure: (a) Fe 2p and (b) S 2p spectra.

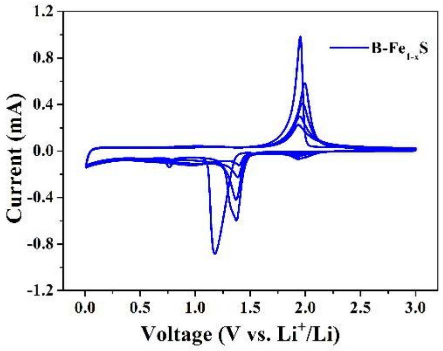

Figure S10. CV curves of B-FeS at a scan rate of 0.1 mV s^{-1}.

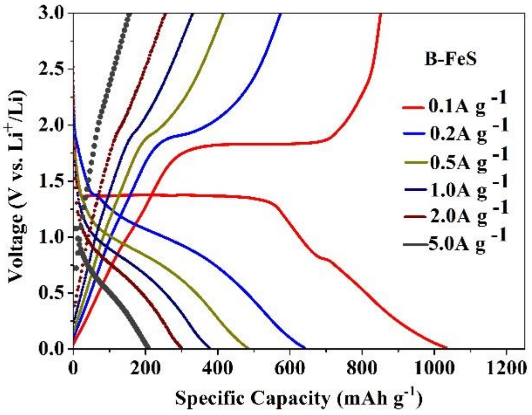

Figure S11. Corresponding galvanostatic discharge/charge at various current densities for B-FeS electrode.

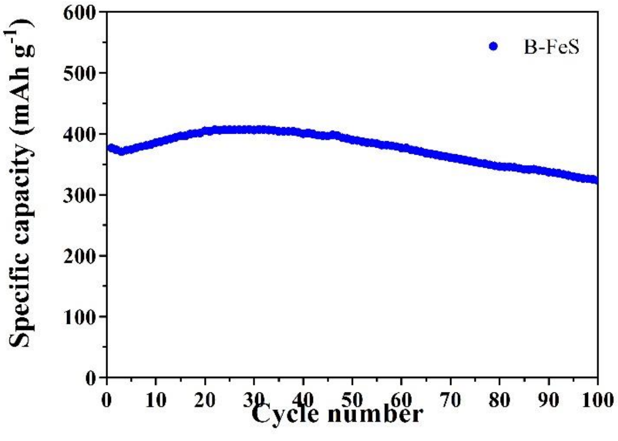

Figure S12. Long-term cyclic performance of B-FeS at the current density of 1.0 A g^{-1}.

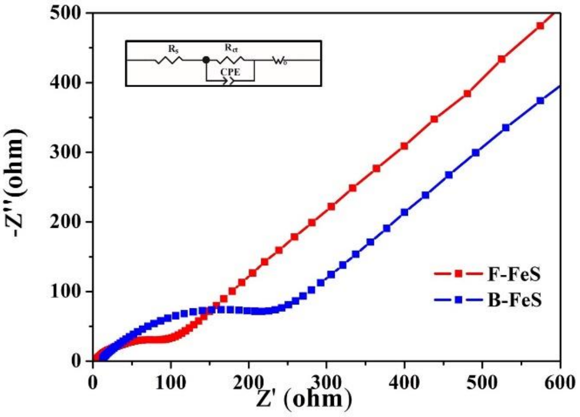

Figure S13. Electrochemical impedance spectra and equivalent circuit of 3D F-FeS and B-FeS nanostructures electrodes after cycling. A sine wave with amplitude of 10.0 mV over the frequency range from 100 kHz to 10 mHz.

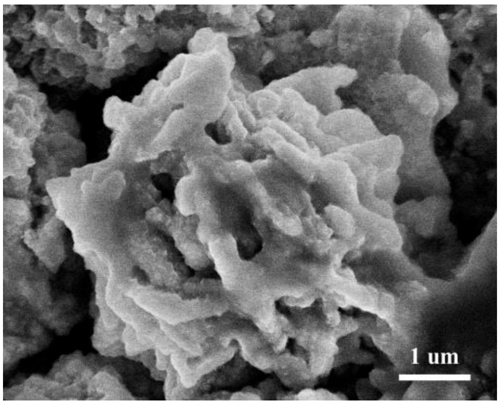

Figure S14. SEM image of 3D F-FeS electrode at full-charge state (3.0 V) after cycling at a current density of 0.1 A g^{-1}.

Table 1. Comparison with previous reports.

electrodes	Current density (A g^{-1})	Capacity (mAh g^{-1})	Initial Coulomic efficiency	references
FS-ND ⊂ PGC-NW	3.0	373.0	79.1%	[18]
FeS@RGO	1.0	200.0	82.0%	[22]
H-FeS@C	2.0	589.0	77.0%	[23]
3D F-FeS	5.0	779.0	78.5%	Our work

Table 2. Impedance parameters obtained using equivalent circuit model for 3D F-FeS and B-FeS nanostructure electrodes.

Table. 2 Impedance parameters obtained using equivalent circuit model for 3D F-FeS and B-FeS nanostructure electrodes

Electrodes	R_s (Ω)	R_{ct} (Ω)	W_o-R
3D F-FeS	0.778	97.89	50.38
B-FeS	2.34	114.7	147.2
